# Comparison of Medicare claims-based *Clostridioides difficile* infection epidemiologic case classification algorithms to medical record review by the Emerging Infections Program using a linked cohort, 2016–2021

**DOI:** 10.1017/ice.2024.204

**Published:** 2025-05

**Authors:** Dustin W. Currie, Chantal Lewis, Joseph D. Lutgring, Sophia V. Kazakova, James Baggs, Lauren Korhonen, Maria Correa, Dana Goodenough, Danyel M. Olson, Jill Szydlowski, Ghinwa Dumyati, Scott K. Fridkin, Christopher Wilson, Alice Y. Guh, Sujan C. Reddy, Kelly M. Hatfield

**Affiliations:** 1 Division of Healthcare Quality Promotion, U.S. Centers for Disease Control and Prevention, Atlanta, GA USA; 2 CDC Foundation, Atlanta, GA, USA; 3 Connecticut Emerging Infections Program, Yale University, New Haven, CT, USA; 4 Georgia Emerging Infections Program, Emory University School of Medicine, Atlanta, GA, USA; 5 New York Emerging Infections Program, University of Rochester Medical Center, Rochester, NY, USA; 6 Tennessee Emerging Infections Program, Tennessee Department of Health, Nashville, TN, USA

## Abstract

**Background:**

Medicare claims are frequently used to study *Clostridioides difficile* infection (CDI) epidemiology. However, they lack specimen collection and diagnosis dates to assign location of onset. Algorithms to classify CDI onset location using claims data have been published, but the degree of misclassification is unknown.

**Methods:**

We linked patients with laboratory-confirmed CDI reported to four Emerging Infections Program (EIP) sites from 2016–2021 to Medicare beneficiaries with fee-for-service Part A/B coverage. We calculated sensitivity of ICD-10-CM codes in claims within ±28 days of EIP specimen collection. CDI was categorized as hospital, long-term care facility, or community-onset using three different Medicare claims-based algorithms based on claim type, ICD-10-CM code position, duration of hospitalization, and ICD-10-CM diagnosis code presence-on-admission indicators. We assessed concordance of EIP case classifications, based on chart review and specimen collection date, with claims case classifications using Cohen’s kappa statistic.

**Results:**

Of 12,671 CDI cases eligible for linkage, 9,032 (71%) were linked to a single, unique Medicare beneficiary. Compared to EIP, sensitivity of CDI ICD-10-CM codes was 81%; codes were more likely to be present for hospitalized patients (93.0%) than those who were not (56.2%). Concordance between EIP and Medicare claims algorithms ranged from 68% to 75%, depending on the algorithm used (κ = 0.56–0.66).

**Conclusion:**

ICD-10-CM codes in Medicare claims data had high sensitivity compared to laboratory-confirmed CDI reported to EIP. Claims-based epidemiologic classification algorithms had moderate concordance with EIP classification of onset location. Misclassification of CDI onset location using Medicare algorithms may bias findings of claims-based CDI studies.

## Background

*Clostridioides difficile* infection (CDI) is one of the most commonly reported healthcare-associated infections in the United States and is associated with substantial morbidity and mortality.^1–4^ CDI surveillance has identified increases in community-associated CDI.^1,5^ Classifying CDI based on the location of onset and potential healthcare exposure is important in understanding transmission patterns, risk factors, and the impact of infection prevention and control measures.

Medicare provides nearly universal healthcare coverage for adults ages 65 and older in the United States,^6–8^ and Medicare claims have been used to understand CDI burden, risk factors, and outcomes.^3,4,9–13^ Use of Medicare claims allows for longitudinal assessment of risk factors and outcomes. However, categorizing CDI cases based on onset location and previous healthcare exposures using claims data can be challenging due to a lack of important data elements (laboratory results and symptom onset, specimen collection, and diagnosis dates).

Algorithms for CDI epidemiologic classification using Medicare claims data have been used.^11,12^ These algorithms are based on claim type and differentiate cases with an ICD-10-CM code of CDI during an inpatient stay by hospital or community-onset categories largely based on either present-on-admission (POA) indicators, which became available in 2011, or the positioning of the CDI diagnosis code and hospital length-of-stay. The potential for misclassification using these different algorithms is largely unknown. One study comparing POA-based CDI onset classification to chart review found moderate sensitivity and high specificity for hospital-onset CDI. However, this study was based on data from a single medical center and used electronic health records rather than claims administrative data.^12,14^ The purpose of our study was to compare Medicare claims-based CDI case classification algorithms to medical chart review using a linked cohort of Medicare beneficiaries and Centers for Disease Control and Prevention’s (CDC’s) Emerging Infections Program (EIP) case-patients.

## Methods

### Study population

We used population- and laboratory-based CDI surveillance data collected by EIP from 2016 through 2021 in selected counties within four geographic catchment areas: Georgia (GA), Connecticut (CT), New York (NY), and Tennessee (TN).^15^ EIP staff aim to identify every CDI case that occurs in residents of the surveillance area using *C. difficile* test results from clinical, reference, and commercial laboratories. An incident EIP CDI case is defined as a positive *C. difficile* molecular or toxin assay in a catchment-area resident aged ≥1 year who did not have a positive test in the prior 8 weeks. For each incident case reported, a basic medical record review is completed for all cases (CT, NY, TN) or for a random sample of cases (GA). Comprehensive record reviews are then completed on all cases determined to be community-onset based on the initial review and on a random sample of healthcare facility-onset cases.

### Data linkage

The first incident case of CDI reported to EIP within a calendar year for each patient aged ≥65 years at specimen collection was eligible for linkage using Medicare claims data. Linkage was only attempted for patients who had a basic medical record review completed by EIP staff (ie, epidemiologic classification was determined).

Medicare beneficiaries were identified in catchment areas for each year based on information contained in their Medicare beneficiary summary files by the state and county. Potential linkages were created by matching Medicare beneficiaries with the same residence area, date of birth, and sex as each EIP case. Potential linkages were scored by having corresponding county of residence and zip codes included on the EIP case report form (CRF) and the Medicare beneficiary summary files.

For each potential EIP-Medicare linkage with CDI-related hospitalization information in the EIP CRF, we used MedPAR hospitalization claims to identify at least one marker of a corresponding hospitalization using admission and discharge dates and facility identifiers. Otherwise, potential EIP-Medicare linkages with prior overnight healthcare exposure in the past 90 days reported in the EIP CRF required at least one corresponding healthcare discharge date and facility identifier from MedPAR skilled nursing facility or acute care hospitalization claims or an overlapping stay reported in the Minimum Dataset 3.0.^16^ For EIP cases without any current or previous healthcare exposures recorded on the CRF, the potential linkage had to have a matching residential zip code. The EIP case was reported as having no linkage if no potential Medicare beneficiaries met these requirements. When there were multiple beneficiaries linked to an EIP case, the Medicare beneficiary with the highest number of corresponding matching criteria was selected as the unique linkage; if a unique beneficiary could not be identified, the EIP case was reported as having multiple linkages.

### Inclusion criteria

All EIP cases without a unique beneficiary linkage were excluded. Next, linked cases that did not have fee-for-service Medicare A and B coverage from the month before through the month after positive specimen collection were excluded. In the case of patient death within the month following specimen collection, only coverage the month before positive specimen collection was required.

### CDI case identification in claims data

For all EIP case-patients meeting the inclusion criteria, we pulled claims from outpatient, carrier, and MedPAR inpatient/skilled nursing facility (SNF) files associated with their Medicare beneficiary ID.^17,18^ Carrier (non-institutional claims submitted by professional providers) and outpatient (outpatient services performed by institutional providers) files were used to identify community-onset cases. CDI cases were identified using ICD-10-CM codes (A04.7, A04.71, A04.72) for claims within ±28 days of the EIP-positive specimen collection date; 28 days was chosen to exclude previous or subsequent CDI infections that were not likely to correspond to the EIP-reported infection of interest. The index date of CDI infection was considered the specimen collection date for EIP data and the first reported claim date with a CDI ICD-10-CM code within ±28 days of EIP specimen collection date for Medicare data.

### Additional case characteristics

Primary diagnosis code was defined as the diagnosis code in the first position within the claim. Information regarding hospitalization status and length of stay came from Medicare MedPAR claims files. ICD-10-CM codes for primary diagnosis of sepsis used in Algorithm 3 (Table 1) included A41.4, A41.8, A41.89, A41.9, R65.2, R65.20, and R65.21. Data collected via EIP CDI CRF, which were used to compare beneficiaries with and without a CDI diagnosis code in claims, included the location of stool collection, hospitalization status within six days of stool collection, in-hospital mortality or 30-day mortality for long-term care facility (LTCF) residents, and presence of recurrent infection.

**Table 1.** Case categorization definitions—Emerging Infections Program chart review versus Medicare Claims-based Algorithms

Epidemiologic class	Emerging infections program	Claims algorithm 1 – CDI ICD-10-CM code position and hospital length of stay	Claims algorithm 2 – CDI ICD-10-CM code present-on-admission indicator	Claims algorithm 3 – CDI ICD-10-CM code position, hospital length of stay, and present-on-admission indicator
**Community-onset community-associated cases (CA)**	Positive stool specimen collected from a patient in an outpatient setting or within 3 days of patient’s hospitalization, who have no history of a stay in a healthcare facility within the 12 weeks prior to stool specimen collection.	*Community onset:* ICD code of CDI from outpatient or carrier claim, or An inpatient visit^a^ for which: **CDI was the primary diagnosis OR** **The primary diagnosis was diarrhea, abdominal pain, or nausea and CDI was coded in a secondary position OR** **CDI was coded in a secondary position and the hospital length of stay was ≤ 3 days**. *Community associated:* no prior inpatient or LTC stay within 12 weeks before diagnosis.	*Community onset:* ICD code of CDI from outpatient or carrier claim, or An inpatient visit for which the CDI diagnosis code was indicated as present on admission (POA). *Community associated:* no prior inpatient or LTC stay within 12 weeks before diagnosis.	*Community onset:* ICD code of CDI from outpatient or carrier claim, or An inpatient visit for which: CDI was the primary diagnosis OR **The primary diagnosis was diarrhea, abdominal pain, or nausea and CDI was coded in a secondary position OR** **CDI was coded in a secondary position and the hospital length of stay was ≤ 3 days OR** **The primary diagnosis was sepsis and CDI was coded in a secondary position coded as present-on-admission**. *Community associated:* No prior inpatient or LTC stay within 12 weeks before diagnosis.
**Community-onset healthcare facility-associated (CO-HCFA)**	Positive stool specimen collected from a patient in an outpatient setting or within 3 days of patient’s hospitalization, who spent at least one night in a healthcare facility within the 12 weeks prior to stool specimen collection	*Community onset:* ICD code of CDI from outpatient or carrier claim, or An inpatient visit for which: CDI was the primary diagnosis OR The primary diagnosis was **diarrhea, abdominal pain, or nausea and CDI was coded in a secondary position OR** **CDI was coded in a secondary position and the hospital length of stay was ≤ 3 days**. *Healthcare associated:* prior inpatient stay within 12 weeks or LTCF stay within 12 weeks to 4 days before diagnosis, no prior CDI diagnosis.	*Community onset:* ICD code of CDI from outpatient or carrier claim, or An inpatient visit for which the CDI diagnosis code was indicated as present on admission (POA). *Healthcare associated:* Prior inpatient stay within 12 weeks or LTCF stay within 12 weeks to 4 days before diagnosis, no prior CDI diagnosis.	*Community onset:* ICD code of CDI from outpatient or carrier claim, or An inpatient visit for which: CDI was the primary diagnosis OR **The primary diagnosis was diarrhea, abdominal pain, or nausea and CDI was coded in a secondary position OR** **CDI was coded in a secondary position and the hospital length of stay was ≤ 3 days OR** **The primary diagnosis was sepsis and CDI was coded in a secondary position coded as present-on-admission** *Healthcare associated:* Prior inpatient stay within 12 weeks or LTCF stay within 12 weeks to 4 days before diagnosis, no prior CDI diagnosis.
**Long-term care facility onset (LTCFO)**	Patient was residing in a long-term care facility three days prior to specimen collection or positive stool specimen was collected in a long-term care facility	Resides in LTCF when infection occurred based on Minimum Data Set (MDS) or MedPAR Skilled Nursing Facility (SNF) claims, or transfers from LTCF to hospital with (a) ICD code of CDI as principal diagnosis for hospital admission, or (b) CDI diagnosis for hospitalization lasting 3 or fewer days.	Resides in LTCF when infection occurred based on MDS or MEDPAR SNF claims, or transfers from LTCF to hospital with the CDI diagnosis code indicated as present on admission.	Resides in LTCF when infection occurred based on MDS or MEDPAR SNF claims, or transfers from LTCF to hospital with (a) ICD code of CDI as principal diagnosis for hospital admission, or (b) CDI diagnosis for hospitalization lasting 3 or fewer days.
**Hospital-onset (HO)**	Positive stool specimen collected more than 3 calendar days after hospital admission	ICD code of CDI (secondary diagnosis) for inpatient admission lasting more than 3 days.	ICD code of CDI during an inpatient visit that was not coded as present on admission.	ICD code of CDI (secondary diagnosis) for inpatient admission lasting more than 3 days, in which the primary diagnosis was not sepsis with CDI coded as present-on-admission.

a An inpatient visit includes any MedPAR claims with an inpatient claim type categorized as a short-stay or long-stay claim.

### Epidemiologic case classification

Based on the positive specimen collection date and data collected during chart review, EIP categorizes CDI cases into the following groups according to well-established epidemiologic categories: community-onset (CO), hospital-onset (HO), and long-term care facility-onset (LTCFO).^15^ CO cases are further subgrouped as community-onset healthcare facility-associated (COHCFA) and community-associated (CA) based on recent healthcare utilization. We classified CDI among linked beneficiaries with evidence of a corresponding CDI diagnosis into the same categories using a combination of previously published and new claims-based algorithms.^11,12^ Table 1 displays the EIP definitions for each of the CDI case categories, as well as corresponding case definitions using Medicare data.

Case classification in Medicare claims data was based on the first available claim with a CDI ICD-10-CM diagnosis code within ±28 days of the EIP specimen collection date. We applied three claims-based case classification algorithms (Table 1), relying on type of index claim (ie, outpatient, carrier, inpatient, SNF), the order of ICD-10-CM CDI and related diagnosis codes, present on admission (POA) diagnosis indicators, duration of hospitalization (for inpatient claims), and LTCF stay indicators in the Minimum Data Set (MDS) (where applicable). Two of the algorithms were based on previously published literature. The first algorithm uses a combination of primary diagnosis code and duration of hospitalization to distinguish CO and HO-CDI (Table 1, Algorithm 1).^11^ The second algorithm distinguishes CO and HO-CDI primarily based on POA indicators for CDI ICD-10-CM diagnosis codes among beneficiaries with an inpatient claim (Table 1, Algorithm 2).^12^ Finally, we created the third algorithm, which combines pieces of the first two algorithms, based on a post-hoc analysis of misclassification from the first algorithm (Table 1, Algorithm 3).

### Data analysis

Claims data were accessed through the Chronic Condition Warehouse Virtual Research Data Center. We report sensitivity of Medicare claims case identification as the proportion of EIP cases with evidence of CDI in Medicare claims, overall and by EIP epidemiologic classification. Chi-square tests were used to compare beneficiaries with and without evidence of CDI based on information collected via EIP CRFs. For Medicare beneficiaries with evidence of CDI, we assessed concordance of EIP and Medicare claims case classification algorithms by reporting percent agreement and Cohen’s kappa statistic. We depict concordant and discordant classifications of symptom onset location using Sankey diagrams. Analyses were completed using SAS Studio. This study was reviewed and approved by the CDC Institutional Review Board (IRB)[§See 45 C.F.R. part 46; 21 C.F.R. part 56.] and was deemed either non-research or received IRB approval with a waiver of informed consent in EIP sites.

## Results

### Study population and data linkage

**Figure 1.**
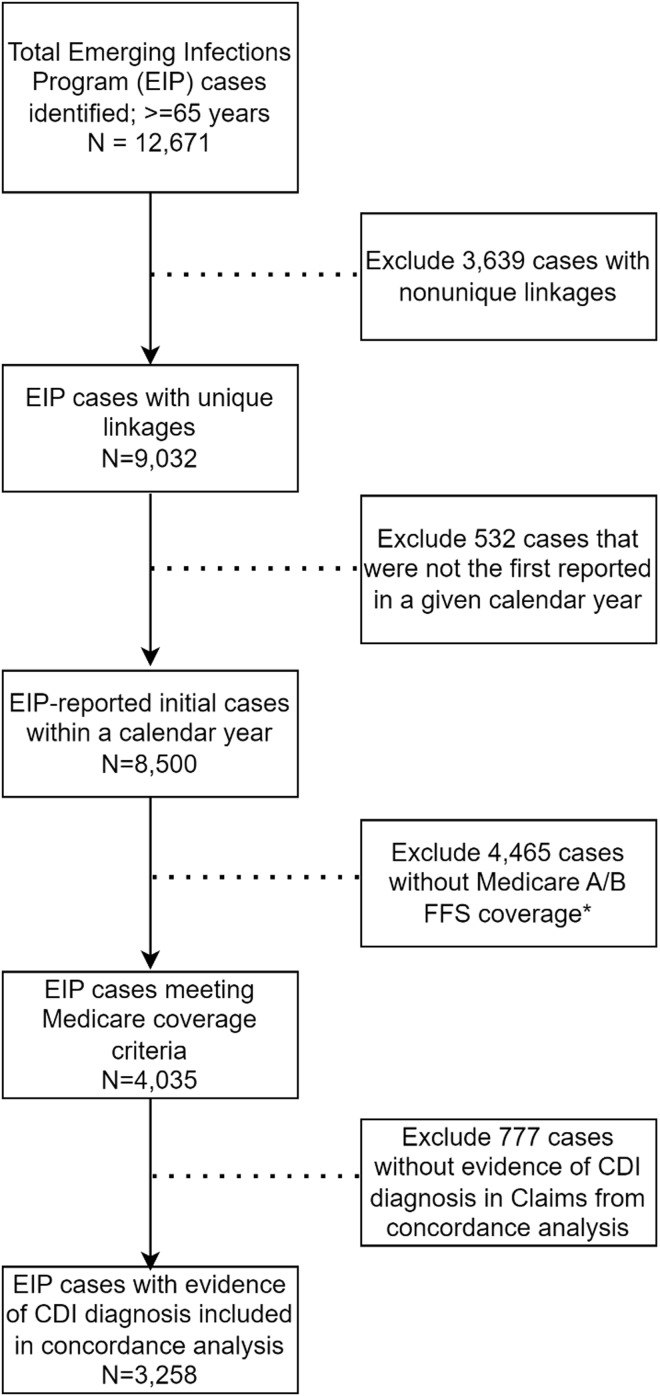
Flowchart depicting study sample after applying inclusion criteria, 4 Emerging Infections Program Sites, 2016–2021. *Coverage criteria include Medicare beneficiaries ages 65 and up with both Part A and Part B fee-for-service (A/B FFS) coverage in the month before through the month after stool collection that tested positive for *Clostridiodes difficile* infection.

**Figure 2.**
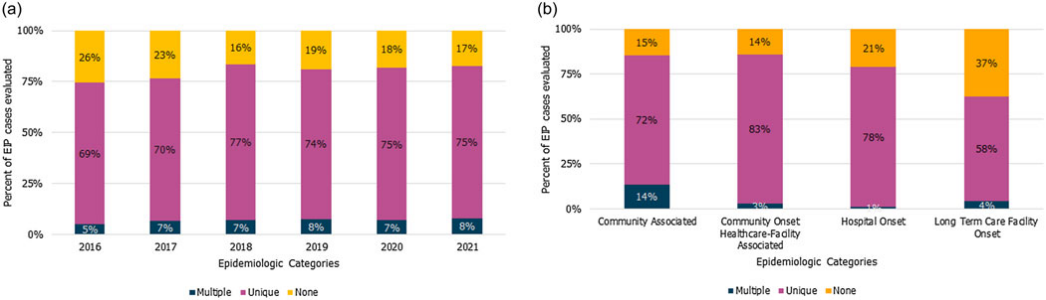
Medicare beneficiary linkage outcome* by Year (2a) and Emerging Infections Program (EIP) *C. difficile* infection epidemiologic classification (2b), 4 EIP Sites, 2016–2021. *A linkage outcome of unique represents a single unique Medicare beneficiary being linked to the EIP case patient. Linkage outcomes of multiple represent EIP case-patients with multiple potential Medicare beneficiaries, and outcomes of none represent EIP case-patients with no potential Medicare beneficiaries using the linkage criteria described.

From 2016 to 2021, a total of 12,671 CDI cases were identified that were eligible for linkage by the four participating EIP sites. Of 12,671 CDI cases eligible for linkage, 9,032 (71%) were linked to a single, unique Medicare beneficiary (Figure 1 and Figure 2a). Success in linkage differed by EIP epidemiologic classification; 83% of COHCFA cases had a successful unique linkage, compared to only 58% for LTCFO (Figure 2b). EIP case-patients with LTCFO CDI represented the highest percentage without a potential linkage (37%), while CA case-patients had the highest proportion linked to multiple potential beneficiaries (14%). After applying the data linkage processes and inclusion criteria, 4,035/12,671 (32%) EIP CDI cases were eligible for analyses.

### Evidence of CDI infection in medicare claims

Of 4,035 eligible EIP CDI case-patients, a CDI ICD-10-CM diagnosis code was present for 3,258 linked beneficiaries (81%, Table 2) within ±28 days of the EIP positive specimen collection date. Lack of CDI diagnosis code was most common for EIP-CA cases (27%) and least common for EIP-HO cases (10%). Location of stool collection, patient hospitalization status, and additional positive *C. difficile* test within 2 and 8 weeks of the incident were all significantly associated with CDI diagnosis code in claims (*P* < .0001 for each; Table 2). Mortality data collected through chart reviews did not differ between those with and without diagnosis codes (*P* = .66).

**Table 2.** Proportion of emerging infections program (EIP)-reported cases with a corresponding *C. difficile* infection ICD-10-CM code in medicare claims among matched medicare beneficiaries by EIP-reported characteristics, 4 EIP Sites,^a^ 2016–2021

EIP-collected case report form information	Total	CDI Identified in Claims N (row %)	CDI not identified in claims N (row %)	*P*-value
Location of stool collection				**<.0001**
Hospital Inpatient	2,248	2,090 (93)	158 (7)	
Emergency Room	234	218 (93)	16 (7)	
LTCF	412	280 (68)	132 (32)	
Outpatient	1,101	636 (58)	465 (42)	
Other (Missing = 13)	27	23 (85)	4 (15)	
Patient hospitalized within 6 days of stool collection				**<.0001**
Yes	2,690	2,503 (93)	187 (7)	
No (Missing = 7)	1,338	752 (56)	586 (44)	
Outcome^b^				.66
Survived	3,680	2,978 (81)	702 (19)	
Died (Missing = 72)	283	232 (82)	51 (18)	
Additional positive *C. difficile* test within 2 and 8 weeks of incident				**<.0001**
Yes	429	372 (87)	57 (13)	
No (Missing = 865)	2,741	2,138 (78)	603 (22)	
EIP Onset Classification^c^				**<.0001**
CO	2,315	1,775 (77)	540 (23)	
HO	1,030	931 (90)	99 (10)	
LTCFO	690	552 (80)	138 (20)	
EIP Case Classification^c^				**<.0001**
CA	1,396	1,020 (73)	376 (27)	
COHCFA	919	755 (82)	164 (18)	
HO	1,030	931 (90)	99 (10)	
LTCFO	690	552 (80)	138 (20)	
Age (years)				**<.0001**
65–74	1540	1188 (77)	352 (23)	
75–84	1437	1183 (82)	254 (18)	
85 and older	1058	887 (84)	171 (16)	
Total	**4,035**	**3,258 (81%)**	**777 (19%)**	

a Participating EIP sites include Connecticut, Georgia, New York, and Tennessee.

b For hospitalized patients, outcome determined at discharge. For non-hospitalized patients that were exclusively seen in the outpatient setting, outcome determined upon leaving the emergency room, observation unit, physician office, or outpatient clinic. For inpatients at a long-term care facility (LTCF) or long-term acute care hospital (LTACH), patient outcome determined at day 30 after specimen collection or upon discharge from LTCF or LTACH, whichever comes first. For patients at a LTCF or LTACH admitted to a short-stay acute care hospital within 30 days after incident specimen collection, outcome determined at hospitalization discharge.

c EIP onset classification categorizes cases into likely location of illness onset (community [CO], hospital [HO], or long-term care facility [LTCFO]). EIP case classification further categorizes community-onset cases into community associated [CA] or healthcare facility-associated [COHCFA] based on whether the patient had a prior inpatient or LTCF stay in the 12 weeks prior to stool collection.

### Epidemiologic class comparisons

Of the 3,258 linked case-patients with evidence of CDI in claims data, concordance in case classification overall was 68% (2203 concordant classifications /3258 case-patients) for Algorithm 1 (κ = 0.56), 75% (2445/3258) for Algorithm 2 (κ = 0.66), and 74% (2398/3258) for Algorithm 3 (κ = 0.64; Table 3).

**Table 3.** Concordance of Emerging Infections Program (EIP) and Medicare claims-based algorithm case classification among patients with *C. difficile* infection identified in claims, 4 EIP sites,^a^ 2016–2021^b^

	EIP Classification					Concordance (κ)
Claims classification	CA	COHCFA	HO	LTCFO	Total	
Algorithm 1						**68% (0.56)**
**CA**	680	55	60	23	**818**	
**Row %**	83.1	6.7	7.3	2.8		
**Column %**	66.7	7.3	6.4	4.2		
**COHCFA**	56	413	53	50	**572**	
**Row %**	9.8	72.2	9.3	8.7		
**Column %**	5.5	54.7	5.7	9.1		
**HO**	267	262	777	146	**1,452**	
**Row %**	18.4	18.0	53.5	10.1		
**Column %**	26.2	34.7	83.5	26.4		
**LTCFO**	17	25	41	333	**416**	
**Row %**	4.1	6.0	9.9	80.0		
**Column %**	1.7	3.3	4.4	60.3		
**Total**	**1,020**	**755**	**931**	**552**	**3,258**	
Algorithm 2						**75% (0.66)**
**CA**	881	76	174	41	**1,172**	
**Row %**	75.2	6.5	14.8	3.5		
**Column %**	86.4	10.1	18.7	7.4		
**COHCFA**	79	614	142	70	**905**	
**Row %**	8.7	67.8	15.7	7.7		
**Column %**	7.7	81.3	15.3	12.7		
**HO**	38	34	533	24	**629**	
**Row %**	6.0	5.4	84.7	3.8		
**Column %**	3.7	4.5	57.3	4.3		
**LTCFO**	22	31	82	417	**552**	
**Row %**	4.0	5.6	14.9	75.5		
**Column %**	2.2	4.1	8.8	75.5		
**Total**	**1,020**	**755**	**931**	**552**	**3,258**	
Algorithm 3						**74% (0.64)**
**CA**	788	69	92	37	**986**	
**Row %**	79.9	7.0	9.3	3.8		
**Column %**	77.3	9.1	9.9	6.7		
**COHCFA**	67	523	66	60	**716**	
**Row %**	9.4	73.0	9.2	8.4		
**Column %**	6.6	69.3	7.1	10.9		
**HO**	146	132	711	79	**1,068**	
**Row %**	13.7	12.4	66.6	7.4		
**Column %**	14.3	17.5	76.4	14.3		
**LTCFO**	19	31	62	376	**488**	
**Row %**	3.9	6.4	12.7	77.0		
**Column %**	1.9	4.1	6.7	68.1		
**Total**	**1,020**	**755**	**931**	**552**	**3,258**	

a Participating EIP sites include Connecticut, Georgia, New York, and Tennessee.

b Concordant classification shaded in light gray. Abbreviations for case classifications are as follow: CA, community-associated; COHCFA, community-onset healthcare facility-associated; HO, hospital onset; LTCFO, long-term care facility onset. Both CA and COHCFA are considered community onset but are differentiated but the patient’s previous healthcare exposure in the prior 12 weeks.

**Figure 3.**
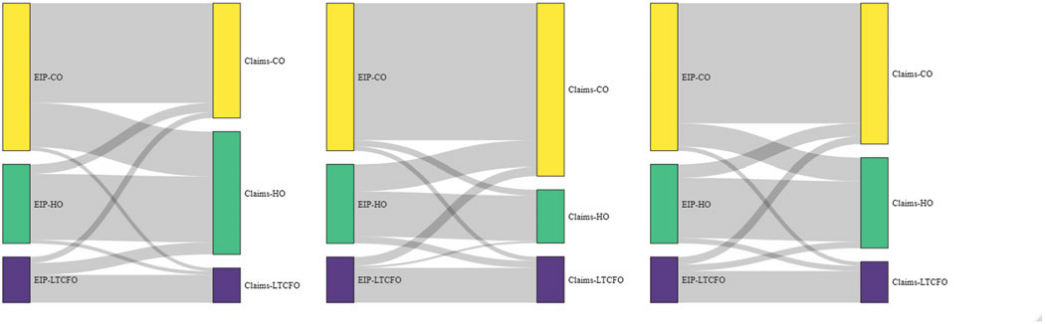
*Clostridiodes difficile* infection (CDI) onset classification using Emerging Infections Program (EIP, left) and claims-based definitions (right). Figure 3a) Algorithm 1, 3b) Algorithm 2, 3c) Algorithm 3. Definitions for EIP as well as each claims-based algorithm provided in Table 1. CDI onset is classified as community-onset (CO), hospital-onset (HO), or long-term care facility onset (LTCFO).

Figure 3a–3c depicts CDI onset classification using EIP and claims-based definitions. Table 3 displays classification concordance with CO cases stratified by healthcare association (eg, CA and COHCFA), while Supplementary Table 1 and Figures 3a–3c present all CO cases together regardless of potential healthcare exposure. The proportion of cases with each EIP onset classification and corresponding claims classifications are shown in gray. Discordant classifications by onset category are displayed in intersecting lines. For Algorithm 1, 30% (529/1775, Supplementary Table 1) of EIP CO cases and 26% (146/552) of EIP LTCFO cases were classified as HO in claims (Figure 3a). HO cases had a higher degree of concordance; over 80% (777/931) of EIP HO cases were also classified as HO using Algorithm 1 (Figure 3a). Of the 529 CDI cases categorized as CO by EIP and HO by the claims algorithm, nearly half had a primary ICD-10-CM diagnosis code of sepsis (252/529; 48%) with CDI listed in a secondary position. This finding was the basis for the creation of Algorithm 3, which uses POA codes for CDI diagnosis when sepsis was the primary diagnosis to further distinguish CO and HO CDI cases.

Algorithm 2, in contrast, more frequently classified EIP HO cases as CO (34%) but had higher concordance for EIP CO cases, 93% of which were classified as CO in claims (Figure 3b). Algorithm 3 had similar levels of discordance across EIP case classifications (Supplementary Table 1); similar proportions of cases were categorized as CO (EIP: 55% [n = 1,775] vs claims: 52% [n = 1,702]), HO (EIP: 29% [n = 931] vs claims: 33% [n = 1,068]), or LTCFO (EIP: 17% [n = 552] vs claims: 15% [n = 488]), despite some discordance in each category.

Table 3 demonstrates the concordance breakdown by both onset location and potential association with prior healthcare exposures in the previous 12 weeks. CDI cases categorized as COHCFA by EIP, in particular, were more likely to be categorized as HO using Algorithm 1 (262/755 [35%]). In general, among CO cases, the claims-based algorithms and EIP agreed on prior healthcare exposure, with relatively few CA cases being misclassified as COHCFA and vice-versa. For example, using Algorithm 1, only 56 EIP-CA cases were categorized as COHCFA, and only 55 EIP-COHCFA cases were categorized as CA.

## Discussion

In our analysis of a linked cohort of Medicare beneficiaries and EIP CDI case-patients, we found evidence of a claims CDI diagnosis code for >80% of cases, indicating relatively high sensitivity for claims to identify the same cases as a population-based laboratory-confirmed surveillance program. Medicare claims-based algorithms to classify cases by onset location and prior healthcare exposure demonstrated moderate concordance with EIP case classification, with one algorithm likely overestimating the proportion of HO cases and one algorithm likely underestimating the proportion of HO cases. A third algorithm developed based on post-hoc analyses of discordance had similar concordance with the two *a priori* defined algorithms, with more balanced discordance across CDI classification categories.

We found high sensitivity (81%) of CDI diagnosis codes in Medicare claims data when compared to EIP cases. This was largely consistent with previous findings within a large healthcare system, which found CDI ICD-9-CM codes within the electronic health records of 88% of true cases (determined via chart review).^12^ Most cases in our study not identified using diagnosis codes in claims data were not hospitalized and had a positive *C. difficile* test collected in an outpatient setting. Differences in case definitions may explain some of the decreased sensitivity of claims data among non-hospitalized patients; EIP’s case definition is laboratory-based and relies on a positive *C. difficile* test. It is possible that some of the cases reported to EIP reflect *C. difficile* colonization rather than infection. In addition, it is possible that some EIP case-patients were linked to an incorrect Medicare beneficiary who did not have CDI, but this is less likely based on our conservative linkage criteria.

Each of the three claims-based algorithms we tested had concordance of 65%–75% regarding location of CDI onset plus potential healthcare exposure. Concordance was higher in the two algorithms that incorporated POA codes. However, the direction of misclassification differed based on the algorithm used. Algorithm 1, based on diagnosis code position and duration of hospitalization for inpatient stays,^11^ had lower concordance overall then the other two algorithms. It correctly identified a very high proportion of cases categorized as HO by EIP, but significant proportions of CO and LTCFO cases were also categorized as HO. Many of the EIP-CO cases misclassified as HO had a primary diagnosis code of sepsis, with a potentially higher severity of illness during hospitalization. Algorithm 2, conversely, had very high agreement with EIP for CO and LTCFO cases, but lower concurrence for HO cases. Algorithm 3 had similar overall concordance to Algorithm 2 but was more balanced across EIP case classification groups. Researchers may use this information to choose the most appropriate algorithm depending on the needs of the specific study and the relative importance of sensitivity for the different case classifications. The relative concordance and lack of directionality in misclassification between EIP and each claims algorithm when comparing CA and COHCFA cases suggest that prior health care exposure can be identified relatively accurately using claims data.

Linking population-based surveillance data with Medicare claims has the potential to improve understanding of risk factors and outcomes of healthcare-associated infections, given the longitudinal nature of claims data as well as more comprehensive surveillance data in the time period surrounding the infection (including laboratory test results, specimen collection information, symptoms, treatment, and index dates). Our linkage procedure, designed to be conservative with a high likelihood of correct linkage at the expense of assigning unique linkages to all cases, successfully linked over 70% of EIP cases to a Medicare beneficiary ID. However, only about half of these beneficiaries met the Medicare A and B fee-for-service coverage criteria. This might be due to a decline in traditional fee-for-service coverage over time as the proportion of beneficiaries enrolling in Medicare Advantage plans has increased.^19^ Innovations in the analysis of Medicare Advantage (Part C) encounter level data may provide further benefits to data linkage. Rates of successful linkage varied by EIP case classification (lowest for patients with LTCFO CDI), which reduced representativeness of linked cohort data compared to population-based representativeness of EIP data. This is likely due in part to most nursing home care not being covered by Medicare. The relative benefits and drawbacks of linking surveillance and claims data should be carefully considered based on the objectives of the specific analysis for which the data will be used.

This analysis is subject to several limitations. Our conservative linkage process resulted in the exclusion of about 30% of EIP cases among adults ages 65 and older due to the lack of a unique linked beneficiary. This prevents accurate assessment of specificity, as lack of a unique linkage may account for a sizeable proportion of CDIs found in claims without an EIP case report. Recurrent CDI was not examined as it is difficult to distinguish primary incident CDI from recurrent or subsequent CDI using claims data. Finally, the COVID-19 pandemic began during our study period, which might have affected CDI diagnostic patterns and onset classifications.

Our study demonstrated high sensitivity of ICD-10-CM CDI codes relative to population-based laboratory surveillance among a linked cohort of Medicare beneficiaries. Epidemiologic case classifications developed using claims-based algorithms had moderate concordance with classifications determined by chart review. Each of the algorithms has specific characteristics and should be applied appropriately based on the objectives of the study. These findings can inform the design of future studies of CDI epidemiology that use administrative claims data for case identification.

## Supporting information

Currie et al. supplementary material Currie et al. supplementary material

## Supplementary material

The supplementary material for this article can be found at https://doi.org/10.1017/ice.2024.204
