# Supplementary material for: Comparison of Medicare claims-based Clostridioides difficile infection epidemiologic case classification algorithms to medical record review by the Emerging Infections Program using a linked cohort, 2016–2021
Source: Infect Control Hosp Epidemiol. 2025 Mar 26;46(5):488–96. doi: 10.1017/ice.2024.204 (PMC12034446; doi:10.1017/ice.2024.204)
Supplement: Currie et al. supplementary material [file S0899823X24002046sup001.docx]

**Supplementary Table 1: Concordance of Emerging Infections Program (EIP) and Medicare Claims Algorithm-based CDI Onset Classification among Patients with CDI Identified in Claims, 4 EIP Sites, 2016-2021.***

|  | **EIP Classification** | | | |
| --- | --- | --- | --- | --- |
| **Claims Classification** | **CO**  **(n=2315)** | **HO**  **(n=1030)** | **LTCFO**  **(n=690)** | **Total**  **(n=4035)** |
| No CDI Diagnosis | | | | |
| **None** | 540 | 99 | 138 | **777** |
| Algorithm 1 | | | | |
| **CO** | 1204 | 113 | 73 | **1,390** |
| **HO** | 529 | 777 | 146 | **1,452** |
| **LTCFO** | 42 | 41 | 333 | **416** |
| **Total** | **1,775** | **931** | **552** | **3,258** |
| Algorithm 2 | | | | |
| **CO** | 1,650 | 316 | 111 | **2,077** |
| **HO** | 72 | 533 | 24 | **629** |
| **LTCFO** | 53 | 82 | 417 | **552** |
| **Total** | **1,775** | **931** | **552** | **3,258** |
| Algorithm 3 | | | | |
| **CO** | 1,447 | 158 | 97 | **1,702** |
| **HO** | 278 | 711 | 79 | **1,068** |
| **LTCFO** | 50 | 62 | 376 | **488** |
| **Total** | **1,775** | **931** | **552** | **3,258** |

*****EIP onset classification categorizes cases into likely location of illness onset (community [CO], hospital [HO], or long-term care facility [LTCFO]).
